# Assessment of nasopharyngeal *Streptococcus pneumoniae* colonization does not permit discrimination between Canadian children with viral and bacterial respiratory infection: a matched-cohort cross-sectional study

**DOI:** 10.1186/s12879-021-06235-z

**Published:** 2021-05-31

**Authors:** Jeffrey M. Pernica, Kristin Inch, Haifa Alfaraidi, Ania Van Meer, Redjana Carciumaru, Kathy Luinstra, Marek Smieja

**Affiliations:** 1 Department of Pediatrics, McMaster University, 1280 Main St. West, Hamilton, Ontario L8S 4K1 Canada; 2 Present address: Department of Pediatrics, King Saud bin Abdulaziz University for Health Sciences, King Abdullah Specialized Children’s Hospital, Ministry of the National Guard Health Affairs, Riyadh, Saudi Arabia; 3 Department of Laboratory Medicine, St. Joseph’s Healthcare Hamilton, 50 Charlton Ave. E, Hamilton, Ontario L8N 4A6 Canada; 4 Department of Pathology and Molecular Medicine, McMaster University, Hamilton, Canada

**Keywords:** Diagnostic microbiology, Pneumonia, Respiratory tract infection, Epidemiology, *Streptococcus pneumoniae*

## Abstract

**Background:**

Readily-available diagnostics do not reliably discriminate between viral and bacterial pediatric uncomplicated pneumonia, both of which are common. Some have suggested that assessment of pneumococcal carriage could be used to identify those children with bacterial pneumonia. The objective of this study was to determine if nasopharyngeal pneumococcal colonization patterns differed between children with definite viral disease, definite bacterial disease, and respiratory disease of indeterminate etiology.

**Methods:**

Three groups of subjects were recruited: children with critical respiratory illness, previously healthy children with respiratory illness admitted to the ward, and previously healthy children diagnosed in the emergency department with non-severe pneumonia. Subjects were categorized as follows: a) viral infection syndrome (eg. bronchiolitis), b) bacterial infection syndrome (ie. pneumonia complicated by effusion/empyema), or c) ‘indeterminate’ pneumonia. Subjects’ nasopharyngeal swabs underwent quantitative PCR testing for *S. pneumoniae*. Associations between categorical variables were determined with Fisher’s exact, chi-square, or logistic regression, as appropriate. Associations between quantitative genomic load and categorical variables was determined by linear regression.

**Results:**

There were 206 children in Group 1, 122 children in Group 2, and 179 children in Group 3. Only a minority (227/507, 45%) had detectable pneumococcal carriage; in those subjects, there was no association of quantitative genomic load with age, recruitment group, or disease category. In multivariate logistic regression, pneumococcal colonization > 3 log copies/mL was associated with younger age and recruitment group, but not with disease category.

**Conclusions:**

The nasopharyngeal *S. pneumoniae* colonization patterns of subjects with definite viral infection were very similar to colonization patterns of those with definite bacterial infection or indeterminate pneumonia. Assessment and quantification of nasopharyngeal pneumococcal colonization does not therefore appear useful to discriminate between acute viral and bacterial respiratory disease; consequently, this diagnostic testing is unlikely to reliably determine which children with indeterminate pneumonia have a bacterial etiology and/or require antibiotic treatment.

## Summary

Our results do not support the use of the assessment and quantification of nasopharyngeal pneumococcal colonization to discriminate between children with viral and bacterial respiratory disease.

## Introduction

Pneumonia is one of the leading causes of paediatric hospitalization in North America. The causes of pneumonia in childhood are many and varied, with viruses, typical bacteria, and atypical pathogens (eg. *Mycoplasma pneumoniae*) all playing important roles [1]. The principles of supportive care for children with respiratory disease, such as the provision of oxygen or ventilatory support, are similar regardless of microbiologic etiology; however, bacterial pneumonia should be treated with appropriate antimicrobials to mitigate disease impact [2]. Consequently, the identification of bacterial respiratory infection is important to facilitate optimal patient management. It is generally accepted that *Streptococcus pneumoniae* is the pathogen that accounts for most severe bacterial community-acquired pneumonia in children, with its identification and treatment of high importance to clinicians [2–4].

Unfortunately, the microbiologic diagnosis of indeterminate (not obviously viral or bacterial) paediatric respiratory infection is not readily accomplished without resorting to highly invasive testing, such as percutaneous trans-thoracic lung aspiration [4]. Nasopharyngeal testing for viruses and atypical pathogens using molecular methods has high sensitivity and specificity [5], but the identification of a viral/atypical pathogen in the nasopharynx does not preclude bacterial co- or super-infection, a common occurrence [1, 6–8]. White blood counts and serum C-reactive protein are weakly predictive of bacterial etiology [2], whereas blood cultures are very specific but insensitive for the detection of bacterial pneumonia [9]. There is substantial inter- and intra-observer variability in the assessment of chest radiographs [10–13], such that their ability to reliably discriminate between bacterial/atypical/viral infection is very limited. Current pneumonia treatment guidelines emphasize that pneumonia in preschoolers often has a viral etiology and does not necessarily warrant treatment [2, 3], but antibiotics are prescribed to most anyway [14]; this may be because clinicians and parents are more concerned about missing a potentially serious bacterial infection than the harms of antimicrobials. As a result, the need for better diagnostics to improve antimicrobial stewardship, even in younger age groups, has been highlighted [15].

Some have recommended measuring pneumococcal nasopharyngeal carriage to discriminate between viral and bacterial infection; cut-offs of 3 log copies/mL [16], 3.9 log copies/mL [17], and 6.9 log copies/mL [18] have been proposed to better identify adults and children with pneumococcal disease. If nasopharyngeal pneumococcal genomic loads were truly higher in children with pneumococcal pneumonia, the most important bacterial pneumonia pathogen [2, 4, 15], this diagnostic test could be used to discriminate between children with primary viral pneumonia and those with bacterial pneumonia, thereby facilitating clinical management and decisions about antibiotic therapy. The objective of this study was to determine if patterns of nasopharyngeal pneumococcal carriage differed amongst children in Canada with definite viral disease, definite bacterial disease, and respiratory disease of indeterminate etiology; it should be emphasized the latter category accounts for the majority of episodes of pediatric community-acquired pneumonia in North America [1, 19, 20].

## Materials and methods

There were three groups of children recruited for this study. Please note that ‘respiratory illness’ was defined as ‘because of perceived respiratory disease (ie. with signs or symptoms of respiratory distress) or requiring either supplemental oxygen or assisted ventilation.’

1. Group 1 consisted of all children aged 2 mos – 18 y admitted to the paediatric intensive care unit (PICU) of McMaster Children’s Hospital (MCH) because of respiratory illness between Sept 2015-Oct 2016 and had a nasopharyngeal swab (NPS) performed less than a week after admission, as previously described, through retrospective review [21].
2. Group 2 consisted of previously healthy children aged 2 mos – 18 y admitted to the general paediatric wards because of respiratory illness from Sept 2015-Jan 2020 prospectively identified through convenience sampling. Those with ventilator-associated pneumonia, cystic fibrosis, anatomic lung disease, bronchiectasis, congenital heart disease, history of repeated aspiration, malignancy, conditions requiring treatment with immune suppressants, or primary immunodeficiency were excluded.
3. Group 3 consisted of previously healthy children aged 6 mos – 10 y who were diagnosed with community-acquired pneumonia in the MCH Emergency Department (ED) and were well enough to be managed as outpatients, as previously described, in a prospective randomized trial [22]. Participants were enrolled during Dec 2012-March 2014 (pilot phase) and Aug 2016-Dec 2019.

Children were categorized into the following disease categories based on discharge diagnoses made by the clinical team and interpretation of chest radiographs made by the attending radiologist:

- Viral infection syndrome (eg. bronchiolitis, asthma exacerbation without a secondary diagnosis of bacterial pneumonia and without a chest radiograph consistent with pneumonia). Current guidelines strongly recommend against treating these children with antimicrobials regardless of illness severity [23, 24].
- Bacterial infection syndrome, ie. pneumonia complicated by effusion/empyema (identified by lung ultrasound).
- ‘Indeterminate’ pneumonia characterized by a clinical diagnosis of pneumonia but without effusion/empyema. Many of these children would have had pure viral infections, but a substantial proportion likely also had bacterial co-infection, as identification of the causative pathogen(s) is generally not feasible [1, 6–8]. As this disease category was of particular interest, it was further subdivided into ‘non-severe’ (ie. did not require hospital admission) and ‘severe’ (ie. was hospitalized).

Please note that the vast majority of children in the ‘viral infection syndrome’ and ‘bacterial infection syndrome’ categories did not require microbiologic confirmation. For example, it is recommended *not* to do microbiologic testing in children with clinically-diagnosed bronchiolitis [23, 24], and the majority of cases of pneumonia complicated by effusion are known to have a bacterial cause [19, 25].

MCH is a tertiary children’s hospital in Hamilton, Ontario, Canada; at the time of the study, the hospital had 259 beds, approximately 6500 admissions, and approximately 40,000 ED visits yearly. All children hospitalized with a potentially infectious respiratory illness at MCH have an NPS performed routinely to identify respiratory viruses, as per the institutional Acute Respiratory Infection Surveillance Protocol.

For each patient, a nasopharyngeal flocked swab (FLOQSwab, Copan Italia, Brescia, Italy) was collected in 3 mL of Universal Transport Medium (UTM, Copan Italia). Samples were stored at negative 80C until testing to preserve sample integrity. After vortexing, 200uL of NPS sample was extracted by easyMag (bioMérieux, St.-Laurent, Quebec, Canada) and eluted in 55uL. Five uL of the eluate was amplified for *S. pneumoniae* using previously published primer and probe sequences [26]. The assay amplifies a 101 bp sequence targeting *lytA*, the autolysin gene. The real-time qPCR assay was run on the Rotor-Gene Q (Qiagen, Germantown, USA) using the QuantiTect Probe PCR Kit (Qiagen). Each reaction contained 5uL of extracted nucleic acid in a final reaction volume of 20uL, 1X QuantiTect Probe PCR Master Mix, 0.4 uM of the forward and reverse primers and 0.1 uM of the TaqMan probe. The probe was labeled with FAM and TAMRA. The assay conditions were 95 °C for 15 min for initial PCR activation followed by 45 cycles of denaturation at 95 °C for 5 s with a combined annealing and extension step at 62 °C for 45 s. Cycle threshold (CTs) values below 40 cycles were considered positive. To verify the specificity of the assay, 20 *S. pneumoniae* positive controls including two ATCC strains, 49619 and 6305 and 18 clinical and laboratory isolates plus 25 negative control organisms, encompassing respiratory organisms and isolates and other streptococcal species were assessed. No cross reactivity was noted with the primers and probes with the viridans group streptococci isolates tested (three different *S. mitis* isolates). In the absence of *S. pseudopneumoniae* strains, the potential cross reactivity of the assay with *S. pseudopneumoniae* could not be ruled out.

Positive samples were further quantified using a standard curve generated from 10-fold serial dilutions of a *lytA* cloned control ranging from 10^2^ to 10^6^ copies/reaction. The cloned control consisted of a 553 bp amplified segment of the *lytA* gene of *S. pneumoniae* ATCC 49619 inserted into a pGEM®-T Easy Vector (Promega, Madison, WI). All generated standard curves had slopes between − 3.4 and − 3.2, and R^2^ values > 0.96 and < 1.

The main outcome was nasopharyngeal pneumococcal genomic load. The covariates of interest were disease category, age (categorized into clinically relevant groups), hospitalization status, and group of recruitment. We did not address potential sources of bias in the analysis, including potential selection bias in groups 2 (ward) and 3 (ED), and did not adjust the analyses for additional potential confounders. Descriptive statistics with appropriate measures of central tendency were presented for all study subjects. Normality was assessed visually. Chi-square or Fisher exact testing, as appropriate, was done to compare categorical variables. Forward logistic regression was done in a stepwise fashion adding covariates found to be significant with *p* < 0.2 in bivariate analyses; those associated with a statistically significant change in the -2log likelihood of the model were retained. No imputation of missing data was done. Analyses were done using STATA v11.2 (College Station, TX). No formal sample size calculation was done in this exploratory study utilizing convenience sampling.

## Results

There were 206 children recruited to Group 1 (PICU), 122 children recruited to Group 2 (hospitalized on ward), and 179 children recruited to Group 3 (ED). The median participant age was 2.5 years (25–75%ile 1.3–4.9 y). There were 160 (32%) with a definite viral infection syndrome, 179 (35%) with non-severe indeterminate pneumonia, 134 (26%) with indeterminate pneumonia who were hospitalized, and 34 (6.7%) with definite bacterial infection (complicated pneumonia). The distribution of the outcome variable of interest, pneumococcal genomic load, was not normal, as a majority of subjects (280/507, 55%) did not have pneumococci detected in their NPS. However, the distribution of genomic load in those with detectable carriage was roughly normal. There were 286 (56%) participants with 0–3 log copies/mL, 153 (30%) with 3–6.9 log copies/mL, and 68 (13%) with > 6.9 log copies/mL in their NPS.

Younger participants were more likely to have detectable pneumococcal carriage. Of those aged < 5 years, 190 (50%) had 0–3 log copies/mL, 127 (33%) had 3–6.9 log copies/mL, and 65 (17%) had > 6.9 log copies/mL in their NPS; in contrast, in those aged 5 and over, 96 (77%) had 0–3 log copies/mL, 25 (20%) had 3–6.9 log copies/mL, and 3 (2.4%) had > 6.9 log copies/mL (*p* < 0.0001). However, among those with *S. pneumoniae* detected, quantitative genomic load was not associated with age (ie. younger children did not have numerically higher pneumococcal loads). Carriage rate also varied by recruitment group; 67% of Group 1 (PICU), 53% of Group 2 (ward), and 42% of Group 3 (ED) did not have *S. pneumoniae* detected (*p* < 0.001). It should be noted, however, that the age distribution of participants was different in different groups. The quantitative genomic load among those with detectable carriage was similar between participants of different groups.

Participants with definite bacterial disease (ie. complicated pneumonia) had very similar colonization patterns as compared to participants with definite viral disease (eg. bronchiolitis) (Table 1). However, it was found that participants with non-severe indeterminate pneumonia had higher carriage rates than participants in other disease categories. In those with detectable carriage, the quantitative genomic load was similar between subjects of all different disease categories.

**Table 1:** Comparison of subjects in different diagnostic disease categories

	Viral infection syndrome (*n* = 160)	Pneumonia, indeterminate, nonsevere (*n* = 179)	Pneumonia, indeterminate, severe (*n* = 134)	Bacterial infection syndrome (*n* = 34)
Age category				
< 2 y	**93 (58%)**	**76 (43%)**	**49 (37%)**	**2 (5.9%)**
2 to <5 y	**36 (22%)**	**65 (37%)**	**43 (32%)**	**18 (53%)**
5 to < 10 y	**22 (14%)**	**36 (20%)**	**26 (19%)**	**8 (24%)**
10 y and over	**9 (5.6%)**	**1 (0.56%)**	**16 (12%)**	**6 (18%)**
Pneumococcus detected?				
Yes	63 (39%)	**103 (58%)**	48 (36%)	13 (38%)
No	97 (61%)	**76 (42%)**	86 (64%)	21 (62%)
Median genomic load (25–75%ile) in those with detectable pneumococcal colonization, log copies/mL	6.12 (4.63–6.89)	6.30 (5.06–7.17)	5.80 (5.30–6.94)	6.26 (5.05–7.63)
Pneumococcal genomic load				
0–3 log copies/mL	98 (61%)	**80 (45%)**	87 (65%)	21 (62%)
3–6.9 log copies/mL	48 (30%)	**61 (34%)**	35 (26%)	9 (26%)
> 6.9 log copies/mL	14 (8.8%)	**38 (21%)**	12 (9.0%)	4 (12%)

Bold indicates differences between categories, *p* < 0.001

Given that age category, recruitment group, and disease category appeared to all be associated with the presence of pneumococcal nasopharyngeal carriage (> 3 log copies/mL), these covariates were entered into the logistic regression model. In this multivariable model, we found that age category continued to exert a dominant effect on the likelihood of colonization; furthermore, the effect of recruitment group was slightly diminished, but was still statistically significant. When age and recruitment group were taken into account, there was no significant residual association between nasopharyngeal pneumococcal carriage and disease category (Table 2).

**Table 2:** Associations with nasopharyngeal pneumococcal colonization > 3 log copies/mL

Covariate	Bivariate analyses		Multivariate analyses	
	OR (95%CI)	Wald p	OR (95%CI)	Wald p
Age category				
< 2 y	ref		ref	
2 to <5 y	1.27 (0.85–1.90)	0.25	1.28 (0.83–1.97)	0.27
5 to < 10 y	0.39 (0.23–0.66)	0.001	0.39 (0.22–0.68)	0.001
10 y and over	0.16 (0.053–0.46)	0.001	0.22 (0.072–0.68)	0.008
Recruitment group				
1 (PICU)	ref		ref	
2 (ward)	1.80 (1.14–2.85)	0.012	1.54 (0.94–2.53)	0.09
3 (ED)	2.62 (1.73–3.98)	< 0.001	2.20 (1.36–3.55)	0.001
Disease category				
Viral infection syndrome	ref		NS	
Nonsevere indeterminate pneumo.	1.96 (1.27–3.02)	0.002		
Severe indeterminate pneumonia	0.85 (0.53–1.38)	0.52		
Bacterial infection syndrome	0.98 (0.46–2.10)	0.96		

*NS* not significant

## Discussion

In this study, we did not find any association between respiratory disease category and *S. pneumoniae* nasopharyngeal carriage > 3 log copies/mL, or pneumococcal quantitative genomic load, when the effects of age and location of recruitment were adjusted for. If quantitation of pneumococcal carriage at admission to hospital could not distinguish between definite viral disease (eg. bronchiolitis) and definite bacterial disease (ie. pneumonia complicated by effusion), it seems unreasonable to posit that this diagnostic testing could be used to determine which children with indeterminate pneumonia had a viral, as compared to bacterial, cause. Our results also do not support the idea of using this diagnostic test as a supplementary diagnostic aid for epidemiologic studies of children presenting with respiratory disease.

Pneumococcal colonization precedes and is presumably a prerequisite for disease caused by *S. pneumoniae* [27]; consequently, it is reasonable to posit that carriage would be more common or nasopharyngeal genomic loads would be higher in those children with pneumococcal infection as compared to those without. This has indeed been demonstrated in multiple different studies of children living in low- and middle-income countries [16, 18, 28–30]. The seminal PERCH case-control study, enrolling in Bangladesh, the Gambia, Kenya, Mali, South Africa, Thailand, and Zambia, found that 56 cases with pneumococcal pneumonia had substantially higher nasopharyngeal pneumococcal genomic loads than 4035 children without pneumococcal disease, with the optimal discriminatory cut-off being 6.9 log copies/mL [18]. One study of 550 consecutive children admitted to a Vietnamese hospital found that those with radiographically-confirmed pneumonia were less likely to be colonized with *S. pneumoniae* than healthy controls; however, those children with pneumonia had higher nasopharyngeal genomic loads than those with other types of respiratory infection or healthy controls [28]. Smaller studies in Mozambique and the Gambia found that paediatric cases with invasive pneumococcal disease or pneumococcal pneumonia had similar colonization rates but higher nasopharyngeal genomic loads than healthy controls [29, 30]. As a result, it has been suggested that the assessment of pneumococcal nasopharyngeal carriage could be used to discriminate between children with and without pneumococcal pneumonia; however, our experience suggests that this may not be true in Canada, and therefore it may not be true in other regions.

The likelihood that *S. pneumoniae* will be detectable in the nasopharynx of a given individual depends on a great number of factors, including age, genetics, socioeconomic conditions, crowding in the home, daycare exposure, and many more [27]. Age is simple to ascertain and appears to play a very prominent role; pneumococcal nasopharyngeal colonization is rapidly acquired during infancy and early childhood and then slowly declines thereafter [27]. In our study, we also observed a clear association between pneumococcal carriage and age. It has also been observed in multiple studies that pneumococcal carriage rates increase with/after respiratory viral infection in children [16, 31–33]. It may be that the combination of these factors play a role in explaining why assessment of nasopharyngeal *S. pneumoniae* colonization did not permit the discrimination of those with bacterial respiratory disease from those with viral disease in our study population.

Our study did have limitations. We did not enroll consecutive children in Cohorts 2 (ward) and 3 (ED), primarily due to resource constraints; these were convenience samples, and so we cannot exclude the possibility of selection bias. We also did not include severity of disease, duration of illness, receipt of antimicrobials prior to nasopharyngeal sampling, sociodemographic factors, daycare exposure, household crowding, genetic factors, vaccination status, and a host of other putative exposures and unmeasured confounders that could possibly influence pneumococcal carriage. Consequently, we cannot exclude the possibility that pneumococcal carriage would in fact be associated with respiratory disease category when these additional factors are taken into account. However, we would suggest that many of these are not practical to measure on a regular basis by clinicians evaluating children with respiratory disease, and so would not impact our recommendations that nasopharyngeal *S. pneumoniae* genomic loads not be routinely used to diagnose bacterial lung infection. In other words, if pneumococcal nasopharyngeal loads were in fact associated with bacterial infection controlling for disease severity, duration of illness, receipt of antimicrobials prior to nasopharyngeal sampling, age, socioeconomic status, number of people in the home, and vaccination status, this would likely not change our conclusion that *S. pneumoniae* nasopharyngeal PCR is not a practical way to diagnose bacterial pneumonia in individual children in an urgent-care setting. Finally, given that diagnoses were made on a clinical basis (as is the standard of care in North America), we could not prove that the proportion of children with viral disease in the ‘viral syndrome’ group was higher than in the ‘indeterminate’ group or the ‘bacterial syndrome’ group; however, this seems exceedingly likely to be true. We would also posit that this consideration is irrelevant, since Canadian physicians routinely make clinical diagnoses without microbiologic confirmation that then inform antibiotic treatment decisions; the aim of this study was to determine if quantitative assessment of nasopharyngeal pneumococcal colonization could aid this diagnostic process, thereby potentially optimizing antimicrobial treatment practices.

## Conclusions

In summary, our matched-cohort study demonstrates that quantitative assessment of nasopharyngeal *S. pneumoniae* carriage is unlikely to be of practical help to clinicians seeking to determine whether a given child has viral or bacterial respiratory infection.

## Data Availability

The datasets used are available from the corresponding author on reasonable request.
